# Epidemiology of suicide among patients referring to Ayatollah Mousavi Hospital in Zanjan between 2016 and 2018

**Published:** 2019-08

**Authors:** Mehran Tahrekhani, Parva Ansari

**Affiliations:** ^*a*^Master of Nursing, Clinical Research Development Center of Ayatollah Mousavi Hospital, Zanjan University of Medical Sciences, Zanjan, Iran.; ^*b*^Ayatollah Mousavi Hospital, Zanjan University of Medical Sciences, Zanjan, Iran.

## Abstract

**Background:**

Suicide is an intentional death which is mainly committed consciously or half-consciously by one’s own intention with the purpose of killing oneself and is one of the most common causes of mortality and morbidity among individuals and many people lose their life annually because of it. Therefore, this study aims to determine the epidemiology of suicide among patients referring to Ayatollah Mousavi Hospital in Zanjan between 2016 and 2018.

**Methods:**

This is a descriptive cross-sectional study. The population of the study included all suicidal patients referred to Ayatollah Mousavi Hospital in Zanjan between 2016 and 2018. Data were collected by referring to the library and the files. The collected data were analyzed using frequency and descriptive statistics and spss software version 20.

**Results:**

The results of this study showed that 253 people have committed suicide, 151 males (59.7%) and 102 females (40.3%) with an average age of 25.66 ± 9.161, of whom 206 (81.4%) were urban and 47 (18.6%) were rural. In terms of marital status, 140 (53.4%) were single and 113 (42.3%) were married. The highest number of reported attempted suicide was 113 (44.7%), between 7 p.m. and 5a.m. Committing self- harm with 119(47.00%) cases were the most common type of suicide and then were taking toxic pills and substances and hanging with 81 (32.01%) and 20 (7.9%) cases respectively. In general, in the second six months of the year, the suicide rate was 55.3%, more than the first six months. Of the total number of suicides, 236 (93.3%) were treated and 17 (6.7%) led to death.

**Conclusions:**

The results of this study showed that suicide attempts were higher in men who were living in urban areas. Therefore, recognizing the causes of suicide and taking preventive measures by authorities and members of the health team can significantly reduce the incidence of suicide.

**Keywords:**

Epidemiology, Suicide, Self-harm

